# Draft Genome Sequence of Vibrio mediterranei Strain CyArs1

**DOI:** 10.1128/mra.00155-22

**Published:** 2022-05-19

**Authors:** João Boaventura, Tânia Caetano, Sónia Mendo, Daniel F. R. Cleary, Newton C. M. Gomes, Vanessa Oliveira

**Affiliations:** a Department of Biology, University of Aveiro, Aveiro, Portugal; b Centre for Environmental and Marine Studies (CESAM), University of Aveiro, Aveiro, Portugal; Montana State University

## Abstract

Here, we report on the draft genome sequence of Vibrio mediterranei strain CyArs1, isolated from the marine sponge *Cinachyrella* sp. Genome annotation revealed multiple genomic features, including eukaryotic-like repeat protein- and multidrug resistance-encoding genes, potentially involved in symbiotic relationships with the sponge host.

## ANNOUNCEMENT

**FIG 1:**
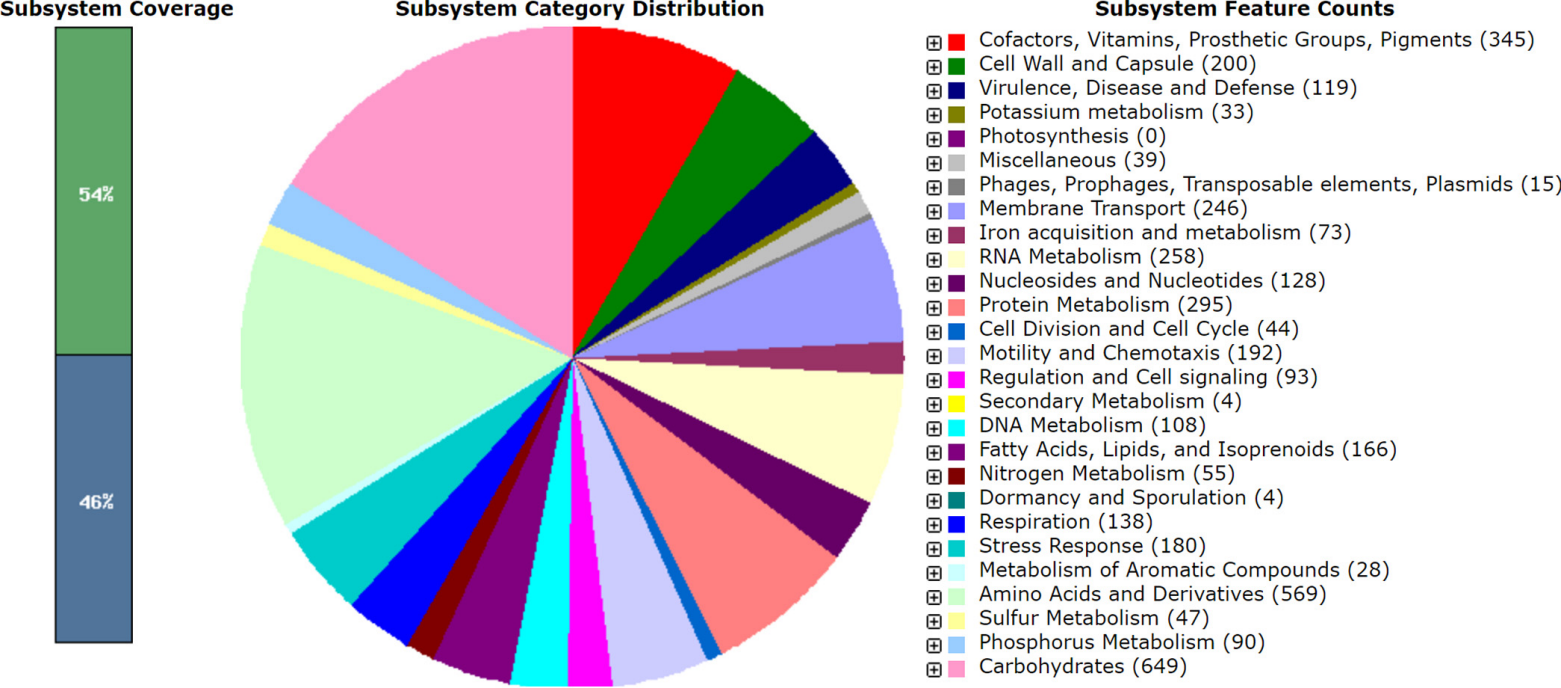
SEED subsystems classification of the Vibrio mediterranei CyArs1 genome based on the RAST annotation server. The subsystem category distribution shows the percentage distribution of the genes in different pathways, which is labeled with different colors, and the number of genes in one feature is mentioned in the brackets.

Vibrio mediterranei has been found in diverse marine environments, such as seawater, sediment, marine invertebrates, bivalves, fish, and red algae (1–4). *V. mediterranei* is considered an opportunistic pathogen and the causative agent of bleaching in the coral Oculina patagonica (1), in addition to being associated with *Pyropia* yellow spot disease (4) and responsible for the greatest mortality in individuals of Pinna nobilis Linnaeus, 1758 (2). *V. mediterranei* strain CyArs1 was isolated from *Cinachyrella* specimens collected in Aimen (Penghu archipelago, Taiwan), after plating host homogenates on half-strength marine agar medium and incubating at 17°C (5). The 16S rRNA gene sequence enabled the identification of the isolate as belonging to the genus *Vibrio*. Genomic DNA was extracted using the FastDNA spin soil kit, from an overnight culture in marine broth supplemented with sodium arsenite (15 μg/μL). The Illumina TrueSeq DNA library preparation kit was employed for library construction, and paired-end sequence reads (2 × 150 bp) were generated on the Illumina HiSeq 2500 platform at Eurofins GATC Biotech (Germany). For all bioinformatics analyses, default parameters were used unless specified otherwise. Adapter sequences and low-quality bases were removed using Trimmomatic v0.39 (6). Trimmed reads were assembled using SPAdes v3.15.2 (–isolate) (7). Draft assembly was filtered by length (<500-bp cutoff), and quality was evaluated using QUAST v5.0.2 (8). Table 1 displays the general features of the *V. mediterranei* CyArs1 genome, which possesses two plasmids of 24,782 bp and 20,920 bp. Isolate CyArs1 was closely related to Vibrio mediterranei (between 97% and 98.36%) based on average nucleotide identity (ANI) values determined using FastANI (9). *V. mediterranei* genome assemblies from GenBank were used for the comparisons. Functional annotation was performed with the Rapid Annotation using Subsystem Technology 2 (RAST 2) server (10). The annotation predicted a total of 5,288 protein-encoding genes, 56 tRNA genes, and 9 rRNA genes (3 5S and 6 16S). Figure 1 revealed the presence of 119 coding DNA sequences (CDSs) classified into the virulence, disease, and defense subsystem, of which 96 CDSs were involved in resistance to antibiotics and toxic compounds, which include genes encoding proteins for multidrug resistance efflux pumps (37 CDSs), resistance to cobalt-zinc-cadmium (23 CDSs), copper (18 CDSs), fluoroquinolones (4 CDSs), arsenic (4 CDSs), beta-lactamase (3 CDSs), bile hydrolysis (3 CDSs), tetracycline (1 CDS), and chromium (1 CDS), as well as multiple antibiotic resistance MAR locus (1 CDSs).

**TABLE 1:** General features of the Vibrio mediterranei CyArs1 genome

Feature	Data
Host species	*Cinachyrella* sp.
No. of reads	7,532,930
Genome size (Mb)	5.72
GC content (%)	44
Genome coverage (×)	300
No. of contigs	46
Contig *N*_50_ (bp)	610,298
Contig L_50_ (bp)	3
Completeness (%)^*a*^	99.9
Contamination (%)^*a*^	2.8
GenBank assembly accession no.	GCA_022432925.1
SRA accession no.	SRP248179
GenBank accession no.	MK533491

a Completeness and contamination were estimated with CheckM v1.1.3 (16).

AntiSMASH v6 (11) identified 6 clusters for secondary metabolites, including (i) two clusters encoding an enzyme normally involved in ribosomally synthesized and posttranslationally modified peptides (RiPPs); (ii) one cluster encoding the biosynthesis of a siderophore homologous to aerobactin, an iron chelating agent produced by Escherichia coli; (iii) one cluster for the biosynthesis of an aryl polyene (APE), similar to the yellow pigment of Vibrio fischeri (12) that is also an antioxidant (13); (iv) one cluster encoding the production of homoserine lactone, an important group of quorum-sensing molecules (14); and (v) one cluster for β-lactone production, which is a chemically diverse group of natural products with high clinical potential (15).

### Data availability.

This whole-genome shotgun project has been deposited at DDBJ/EMBL/GenBank under the accession number JAKRBO000000000. The version described in this paper is the first version, JAKRBO010000000.

All high-throughput sequencing data used in this study are available in the European Nucleotide Archive under accession number PRJNA593396 and in SRA under SRP248179.
